# An Integrative Clinical Model for the Prediction of Pathological Complete Response in Patients with Operable Stage II and Stage III Triple-Negative Breast Cancer Receiving Neoadjuvant Chemotherapy

**DOI:** 10.3390/cancers14174170

**Published:** 2022-08-28

**Authors:** Wai-Shan Chung, Shin-Cheh Chen, Tai-Ming Ko, Yung-Chang Lin, Sheng-Hsuan Lin, Yung-Feng Lo, Shu-Chi Tseng, Chi-Chang Yu

**Affiliations:** 1 Department of Surgery, Chang Gung Memorial Hospital at Linkou, Taoyuan 333, Taiwan; 2 College of Medicine, Chang Gung University, Taoyuan 333, Taiwan; 3 Department of Biological Science and Technology, National Yang Ming Chiao Tung University, Hsinchu 300, Taiwan; 4 Institute of Biomedical Sciences, Academia Sinica, Taipei 115, Taiwan; 5 Department of Medicine, Division of Medical Oncology, Chang Gung Memorial Hospital, Chang Gung University Medical College, Linkou Branch, Taoyuan 333, Taiwan; 6 Institute of Statistics, National Yang Ming Chiao Tung University, Hsinchu 300, Taiwan; 7 Department of Medical Imaging and Intervention, Chang Gung Memorial Hospital at Linkou, 5 Fuhsing St., Guishan, Taoyuan 33382, Taiwan

**Keywords:** triple-negative breast cancer, neoadjuvant chemotherapy, treatment response prediction

## Abstract

**Simple Summary:**

Neoadjuvant chemotherapy (NAC) is widely used to treat stage II and III primary, operable triple-negative breast cancer (TNBC). The response to NAC critically affects the subsequent treatment plan, including not only curative surgical planning but also adjuvant therapy. There is no standard prediction model that accurately predicts NAC response. Therefore, the development of an easy-to-apply and cost-effective clinical prediction model for NAC treatment response would improve clinical practice. We propose an integrative clinical prediction model for the prediction of pathologically complete response in patients with operable stage II and stage III TNBC receiving NAC based on findings from tumor ultrasound and blood tests. All included parameters were readily available during and before NAC. This clinical prediction model could provide a reference to guide clinicians’ decisions in planning a patient’s NAC treatment as early as after the first cycle of NAC.

**Abstract:**

Triple-negative breast cancer (TNBC) is treated with neoadjuvant chemotherapy (NAC). The response to NAC, particularly the probability of a complete pathological response (pCR), guides the surgical approach and adjuvant therapy. We developed a prediction model using a nomogram integrating blood tests and pre-treatment ultrasound findings for predicting pCR in patients with stage II or III operable TNBC receiving NAC. Clinical data before and after the first cycle of NAC collected from patients between 2012 and 2019 were analyzed using univariate and multivariate analyses to identify correlations with pCR. The coefficients of the significant parameters were calculated using logistic regression, and a nomogram was developed based on the logistic model to predict the probability of pCR. Eighty-eight patients were included. Five parameters correlated with the probability of pCR, including the neutrophil-to-lymphocyte ratio, platelet-to-lymphocyte (PLR) ratio, percentage change in PLR, presence of echogenic halo, and tumor height-to-width ratio. The discrimination performance of the nomogram was indicated by an area under the curve of 87.7%, and internal validation showed that the chi-square value of the Hosmer–Lemeshow test was 7.67 (*p* = 0.363). Thus, the integrative prediction model using clinical data can predict the probability of pCR in patients with TNBC receiving NAC.

## 1. Introduction

Neoadjuvant chemotherapy (NAC) is widely used for locally advanced triple-negative breast cancer (TNBC) in the preoperative setting. Locally advanced TNBC with a tumor size > 2 cm with or without ipsilateral axillary node invasion has been recommended for perioperative systematic treatment by several guidelines [1,2]. This concept has been investigated in clinical trials for its efficacy and safety [3,4,5]. Preoperative systematic treatment is aimed at downstaging and guiding postoperative adjuvant treatment and may also improve overall oncological outcomes in primary, operable TNBC [4,6,7]. After cancer downstaging, initially inoperable or mastectomy-only breast cancer has more surgical options, including breast-conserving surgery, which can provide patients with better cosmetic outcomes and quality of life. The response to NAC-guided postoperative adjuvant therapy is important, particularly for those with residual disease requiring escalation of systematic treatment [8].

Pathological complete response (pCR) after NAC has long-term benefits for patients [9,10]. pCR is defined as no residual invasive disease in the breast or axillary lymph nodes. Improved oncological outcomes, such as a lower recurrence rate and better overall survival, were reported in patients with TNBC who achieved pCR after NAC [11]. The pCR rate of NAC in TNBC is approximately 30% to 45% with chemotherapy alone, and the rate is further increased to approximately 65% with chemotherapy combined with immunotherapy [12,13,14]. Research has been conducted to analyze and identify biomarkers to predict pCR, since only a proportion of patients achieve pCR status. Most studies focused on prognostic biomarkers rather than predictive indicators detected before treatment or at the initiation of NAC [15,16,17]. Clinical predictive biomarkers of pCR in TNBC have been reported, but a consensus is not yet well established [18,19,20]. The prediction of pCR or residual disease has important clinical implications for patients receiving NAC.

At the initiation of therapy or in the very early period of treatment, only tumor images, biopsy pathology reports, and blood test information are available clinically. Currently, there is no widely accepted biomarker or model for the prediction of pCR in patients with TNBC receiving NAC based on the integration of a patient’s tumor ultrasound image and blood test findings. We aimed to develop an integrative clinical model for the prediction of pCR in patients with operable stage II or III TNBC receiving NAC.

## 2. Materials and Methods

### 2.1. Patients

**Figure 1:**
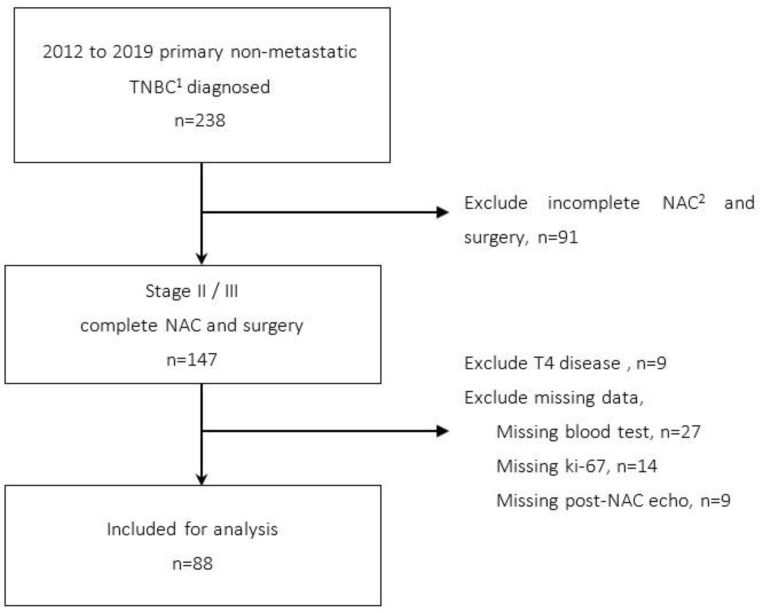
Flowchart of patients recruited in this study. ^1^ TNBC: triple negative breast cancer. ^2^ NAC: neoadjuvant chemotherapy.

Between 2012 and 2019, as shown in Figure 1, a total of 238 female patients were diagnosed with primary non-metastatic TNBC at Linkuo Chang Gung Memorial Hospital. We included only patients diagnosed with stage II to stage III primary TNBC who received NAC followed by surgery (n = 147). NAC regimens included 4 cycles of anthracycline with cyclophosphamide every 3 weeks and 4 cycles of taxane with platinum every 3 weeks given sequentially. Patients with missing blood test data before the first cycle of NAC (n = 27), incomplete pathology reports in which either Ki-67% or tumor grade were not reported (n = 14), T4 disease where ultrasound assessment was difficult (n = 9), or without post-NAC ultrasound examination (n = 9) were excluded. Therefore, 88 patients were included in the final analysis. According to the AJCC staging guideline, T4 disease was defined as a tumor of any size with direct extension to the chest wall and/or to the skin (ulceration or skin nodules) in which the assessment of tumor size by ultrasound was difficult. Due to the limitation of ultrasound assessment for T4 disease, patients with T4 disease were excluded. This study was approved by the Institutional Review Board of Linkuo Chang Gung Memorial Hospital (IRB approval number: 202101328B0). 

### 2.2. Parameters

Clinicopathological data, ultrasound examination, and blood test results were prospectively recorded after the initial diagnosis of breast cancer. Data were collected from electronic medical records.

Clinicopathological data, which were determined based on the American Society of Clinical Oncology/College of American Pathologists (ASCO/CAP) guidelines, including the expression of estrogen receptors, progesterone receptors, human epidermal growth factor receptor 2 (HER2), Ki-67%, and tumor histology, were obtained from the initial breast tumor biopsy. Tumor grading, tumor size, and axillary lymph node invasion were obtained from surgical resections post-NAC. The pathological stage of the residual disease was determined according to the 8th American Joint Committee on Cancer (AJCC) guidelines. pCR was defined as the absence of residual invasive tumor cells in the breast and axillary lymph node specimens obtained by surgical excision after NAC.

Ultrasound images were reviewed individually by two breast ultrasound specialists. Recorded lexicons, including tumor height, width, length, shape, margin, orientation, echo pattern, posterior features, and other associated features, were collected during both pre- and post-NAC ultrasound examinations. The tumor height-to-width (H/W) ratio was calculated using data from the pre-NAC ultrasound examination.

Blood test data, including white blood cell count, neutrophil count, lymphocyte count, platelet count, monocyte count, red blood cell count, hemoglobin, and mean corpuscular volume, were collected before initial NAC and after the 1st cycle of NAC. The neutrophil-to-lymphocyte ratio (NLR) and platelet-to-lymphocyte ratio (PLR) were then calculated. The changes in NLR and PLR were defined as follows:

$$\mathrm{Change}\;\mathrm{in}\;\mathrm{NLR}=\frac{\mathrm{NLR}\;\mathrm{after}\;\mathrm{the}\;1\mathrm{st}\;\mathrm{cycle}\;\mathrm{NAC}\;-\;\mathrm{NLR}\;\mathrm{before}\;\mathrm{the}\;1\mathrm{st}\;\mathrm{cycle}\;\mathrm{NAC}}{\mathrm{NLR}\;\mathrm{before}\;\mathrm{the}\;1\mathrm{st}\;\mathrm{cycle}\;\mathrm{NAC}}$$

$$\mathrm{Change}\;\mathrm{in}\;\mathrm{PLR}=\frac{\mathrm{PLR}\;\mathrm{after}\;\mathrm{the}\;1\mathrm{st}\;\mathrm{cycle}\;\mathrm{NAC}\;-\;\mathrm{PLR}\;\mathrm{before}\;\mathrm{the}\;1\mathrm{st}\;\mathrm{cycle}\;\mathrm{NAC}}{\mathrm{PLR}\;\mathrm{before}\;\mathrm{the}\;1\mathrm{st}\;\mathrm{cycle}\;\mathrm{NAC}}$$

### 2.3. Statistical Analyses

Age was analyzed as a continuous variable, whereas T stage, tumor grading, echogenic halo sign, and tumor posterior features were analyzed as categorical variables. Ki-67%, NLR, PLR, NLR change, and PLR change were divided into two groups according to the cutoff points calculated by maximizing the (sensitivity + specificity) point of the receiver operating characteristic (ROC) curve for the prediction of pCR, as there is no definite cutoff for the above parameters in TNBC.

The correlations between pCR and age were analyzed using a Wilcoxon rank-sum test, while chi-square tests were used for all other parameters. Univariate and multivariate analyses using forward stepwise logistic regressions were performed to identify predictive factors for pCR after NAC. The standard coefficients of the individual parameters were calculated using logistic regressions, and a nomogram was developed based on the logistic model. The nomogram calibration by internal validation using the bootstrap resampling approach was then displayed using a calibration curve. The Hosmer–Lemeshow test was employed to measure the goodness of fit for the model. The discrimination of the nomogram was plotted using an ROC curve, and the area under the curve (AUC) was quantified. Statistical analyses were performed using R software (R Foundation for Statistical Computing, Vienna, Austria, version 3.3.3). The statistical significance was set at *p* < 0.05.

## 3. Results

### 3.1. Clinical Characteristics of Patients

A total of 88 female patients diagnosed with primary non-metastatic TNBC completed NAC and underwent surgery. Complete data, including pre-treatment ultrasound images, pre-NAC blood test data, post-first cycle NAC blood test, post-treatment ultrasound image, and comprehensive pathological report for analysis, were available for all patients.

The clinicopathological characteristics of patients before and after NAC in this study cohort are summarized in Table 1. Among all patients, 30.7% (n = 27) achieved pCR, while 69.3% did not (non-pCR). The mean age was 50.6 ± 10.0 for the pCR group compared to 50.9 ± 10.4 for the non-pCR group. Most of the patients were at the clinical T2 stage, with 88.9% (n = 24) in the pCR group and 70.5% (n = 43) in the non-pCR group. The optimal cutoff value of the Ki-67 index was 34%, the NLR was 1.91, the PLR was 148.14, the percentage NLR change was −0.165, the percentage PLR change was 0.038, and H/W ratio was 1.22.

### 3.2. Factor Predicting pCR for Patients Receiving NAC

To further analyze the influence of patients’ pathological and blood test values as well as their ultrasound characteristics on the probability of pCR, a univariate logistic regression was performed (Table 2). High Ki-67% (*p* = 0.034), high tumor grade (*p* = 0.024), lower percentage change in PLR (*p* = 0.004), presence of echogenic halo (*p* = 0.001), and high H/W ratio (*p* = 0.47) were related to the probability of pCR. We then conducted multivariate analysis using forward stepwise logistic regressions to identify the features that can help predict pCR (Table 2). Five features were related to the prediction of pCR: NLR (*p* = 0.01), PLR (*p* = 0.01), percentage change in PLR (*p* = 0.02), echogenic halo (*p* = 0.002), and H/W ratio (*p* = 0.025). Furthermore, the estimated coefficients of each predictive feature were analyzed using logistic regressions, in which the absence of echogenic halo, large NLR, and large percentage change in PLR negatively influenced the probability of pCR, while large PLR and high H/W ratio were inversely correlated (Table 2).

### 3.3. Model for the Prediction of pCR in Patients Receiving NAC

**Figure 2:**
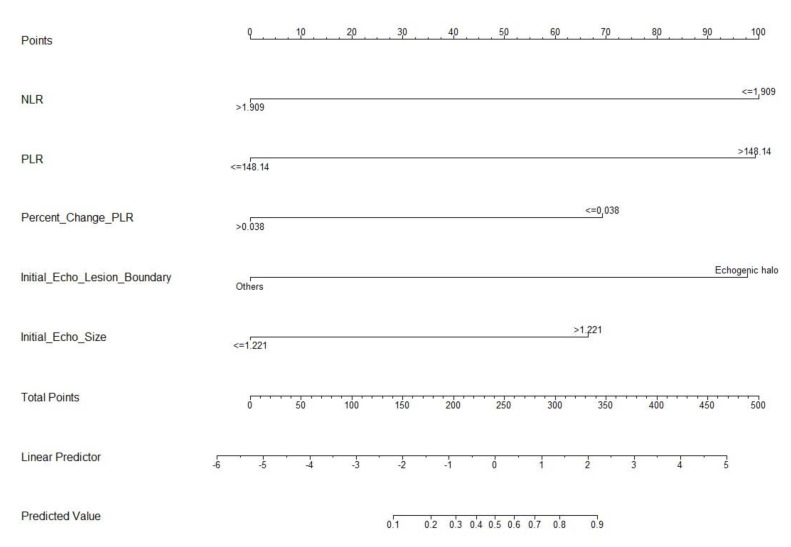
Nomogram for prediction of pCR in TNBC patients receiving NAC. NLR: neutrophil-to-lymphocyte ratio. PLR: platelet-to-lymphocyte ratio. Initial_Echo_Size: H/W ratio (tumor height-to-width ratio).

**Figure 3:**
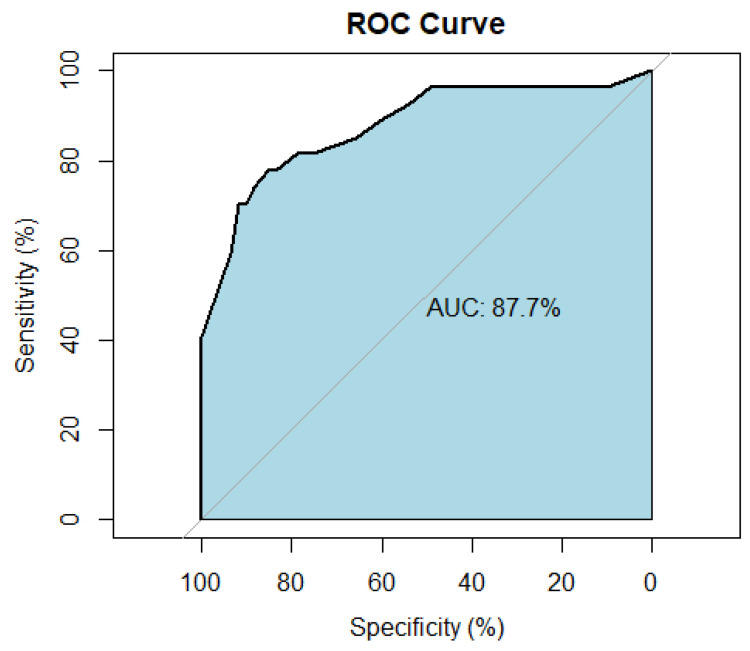
The ROC curve with an AUC of 0.877 to demonstrate the discriminatory ability of the nomogram for predication of pCR in TNBC patients receiving NAC. ROC: receiver operating characteristic (ROC) curve. AUC: area under the curve, representing the discrimination of the nomogram.

All five significant features (NLR, PLR, percentage change in PLR, presence of echogenic halo, and H/W ratio) identified by multivariate analysis were applied to develop a nomogram for predicting pCR in patients with TNBC receiving NAC (Figure 2). To determine the probability of pCR, the points of each feature were summed to obtain the total points. By referencing the total points vertically to the predicted value, the individualized probability of pCR can be identified. As shown in Figure 2, the NLR and PLR had the greatest impact on the probability of pCR, followed by the presence of an echogenic halo. The percentage change in PLR and the H/W ratio had less impact on the probability of pCR. The performance of our nomogram discrimination is shown in Figure 3, suggesting that it is a good prediction model with an AUC of 87.7% in the ROC curve.

**Figure 4:**
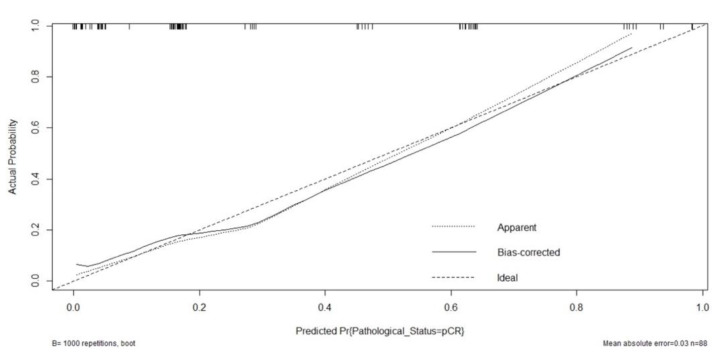
The calibration curve is based on internal validation with a bootstrap resampling frequency of 1000.

Internal validation by the bootstrap resampling procedure (B = 1000) is presented by the calibration curve using the Hosmer–Lemeshow goodness-of-fit test to show the consistency of the true probability and the estimated probability (Figure 4). Our model has an optimal agreement between the predictions by the nomogram and the actual observations, with the chi-square value of the Hosmer–Lemeshow test being 7.67 (*p* = 0.363).

To apply this nomogram to an individual patient, we first read the first row of the nomogram (points) and assigned points for all five variables. Then, the sum of all the points and the total was found in row seven (total points). Finally, the total points in row seven were located, and a vertical line between the total points and row eight (predicted value) was used to obtain the probability of pCR. For example, a 58-year-old female had a 3.23 × 4.58 × 1.26 cm TNBC tumor, present echogenic halo, 40% Ki-67, an initial NLR of 0.61, a PLR of 158, and a PLR change of 0.04. To apply this nomogram to prediction, we first read the assigned points for the five variables. The points for NLR, PLR, and the echo boundary were 100, and those for PLR change and echo size were 0. The total points were 300, so the predicted probability of pCR was approximately 80%.

## 4. Discussion

To the best of our knowledge, this is the first study to develop an integrative clinical prediction model for the prediction of pCR in patients with operable stage II and III TNBC receiving NAC based on tumor ultrasound findings and blood tests. Pre-treatment breast ultrasound provided direct features of the tumor. Blood tests at pre-treatment and after the first cycle of chemotherapy provided indirect information on the patient’s tolerance of systemic therapy. Integration of these two widely available clinical data points helped to assess the probability of the final outcome, pCR or residual disease, after completing NAC within the first months of treatment initiation. Furthermore, this model may be used to assess treatment responsiveness in the early stages of future clinical trials or novel preoperative systemic therapy.

The prediction of pCR in patients receiving NAC has long been an interesting topic in the oncology community. Studies have shown the predictive power of NAC in patients with TNBC [21,22]. However, most of these studies focused on the analysis of data from blood tests only, with little focus on the dynamic change of patients’ response to NAC. Our model combines not only pre-treatment and dynamic blood test information but also another category of tumor imaging features. Images, especially breast ultrasound, are crucial for pre-treatment studies and in most patients are used as diagnostic images.

Ultrasonography is an important tool for the assessment of breast cancer during diagnosis and preoperative assessment. In patients receiving NAC, ultrasound can also be used to assess treatment response. Compared with other imaging tools for NAC treatment response assessment, such as MRI, ultrasound is less costly, less complex, and more accessible. Studies have shown the accuracy and reliability of NAC response assessments for guided individualized treatment [23,24,25]. The presence of an echogenic halo sign refers to the hyperechoic zone around the tumor, which is thought to be the displacement of surrounding tissue and perifocal edema, tumor infiltration, and neo-vascularization related [26,27]. The echogenic halo sign may indicate a high proliferative index in the tumor, which is related to better chemotherapeutic sensitivity [28,29]. The H/W ratio represents an overview of the tumor shape and axis. A high H/W ratio refers to a taller-than-wide tumor with non-parallel features.

Systemic inflammation has been widely investigated in oncology [30,31]. Some inflammatory-based scores, such as NLR, PLR, change in NLR, and change in PLR, are related to breast cancer prognosis and treatment outcome [31,32,33,34,35]. In our model, low NLR, high PLR, and a lower percentage change in PLR were associated with pCR. NLR represents the systemic immunoreaction in which neutrophils inhibit the immune system and promote tumor growth by suppressing the activity of lymphocytes and T-cell responses [36]. A low NLR indicates a tumor suppressive effect by the inert immune system, leading to pCR, which has also been suggested by other studies [37,38]. PLR represents cancer cell activity, and platelets are related to platelet-derived growth factors [39,40]. Small studies have shown that PLR may predict the response to NAC in breast cancer [41,42]. Thus, the combination of NLR and PLR at pre-treatment helps predicts pCR in NAC [43].

For patients with breast cancer treated with NAC, pCR is considered a primary endpoint. pCR was approved by the US Food & Drug Administration as a surrogate endpoint and was the basis of drug approval or licensure mandated by Section 507 of the FD&C Act [44]. pCR is considered an important endpoint in NAC treatment because patients with breast cancer who achieve pCR after NAC have improved disease-free survival, especially among the molecular subtypes of TNBC [10]. Furthermore, in long-term follow-ups, groups with no residual tumors after NAC demonstrated a significantly better overall survival than those with residual disease [45]. Breast-conserving surgery becomes more feasible after NAC [46]. This benefits female patients with breast cancer by providing more surgical options that can strike a balance between satisfactory oncological outcomes and body image integrity. Among the different molecular subtypes of breast cancer, TNBC (basal-like subgroup) was observed to have a better response and higher pCR rate to NAC with preoperative paclitaxel, followed by 5-fluorouracil, doxorubicin, and cyclophosphamide chemotherapy [47]. In contrast to pCR, residual cancer burden negatively affects oncological outcomes, including higher invasive disease recurrence and worse overall survival [48]. A clinical trial was performed to counteract the adverse prognosis by escalating adjuvant treatment for patients who failed to achieve pCR with NAC [49]. In summary, pCR plays a critical role in the perioperative treatment of breast cancer. Therefore, prediction of pCR facilitates the identification of high-risk patients requiring escalation treatment in the early phase and also supports decision-making between clinicians and patients.

Our nomogram contained five variables, including two ultrasound parameters: presence of an echogenic halo and H/W ratio, and three blood test results: NLR, PLR, and percentage change in PLR. Except for the percentage change in PLR, which was collected after the first cycle of NAC and marked the dynamic change in the patient’s immune response, the other two blood test parameters, NLR and PLR, were collected before treatment. This effectively integrates the tumor presentation in the image and the patient’s immune system before and in response to systemic treatment with NAC. These findings represent a simple and visual tool for the prediction of pCR in patients with operable stage II and III TNBC receiving NAC.

This model can assist clinicians in predicting treatment response in terms of pCR in patients with TNBC undergoing NAC as early as after the first cycle of therapy. This helps treatment escalation modification, which can be made earlier to provide better patient care and improve patients’ overall oncological outcomes. In the future, the prediction model can be employed in clinical trial candidate surveys to identify patients who are expected to have a poor response to standard NAC for TNBC. Thus, our prediction model is significant for both current and future applications.

Our study had certain limitations. First, this was a retrospective study, and the results may have been affected by data collection bias. We carefully read every medical report during data collection, and each ultrasound sound image was reviewed by two individual breast ultrasound specialists to minimize data collection bias. Second, the number of cases included in the final analysis was relatively small and the calibration of our prediction model was statistically based on internal validation. Although the sample size was limited, the training set used to develop the nomogram comprised all the patients, which is a generally accepted method for nomogram construction and validation. The internal calibration of accuracy is a statistically reliable method for the assessment of a prediction model, particularly for a small dataset. Further external validation based on other populations is needed to estimate model accuracy. Third, TNBC is a heterogeneous disease that includes several subtypes of tumors (luminal androgen receptor, mesenchymal, basal-like, and others). There are differences in prognosis among the different subtypes of TNBC. In addition, many other factors may be related to the response to NAC, such as BRCA mutation status, race, and menopausal status. It is possible that taking these factors into account will further improve the accuracy of the prediction. However, a user-friendly and effective clinical prediction model should be as simple as possible and still provide a satisfactorily accurate estimation. Thus, our model included only routinely collected clinical parameters to help predict the probability of pCR in patients with TNBC receiving NAC treatment.

## 5. Conclusions

In conclusion, we developed a clinical prediction model for pCR in patients with operable stage II and III TNBC receiving NAC by integrating parameters from breast ultrasounds and blood tests routinely collected in daily clinical practice. The nomogram is a reliable and easy-to-use clinical prediction model. This preliminary result feasibly helps clinicians plan treatment in the early period after preoperative systemic treatment initiation and share decision making with patients.

## Figures and Tables

**Table 1:** Demographic data of patients.

Characteristic	Non-pCR (n = 61)	pCR (n = 27)
Age		
Mean (SD) ^1^ Median [min.,max.]	50.9 (10.4)	50.6 (10.0)
	51 [21.0,79.0]	51.0 [33.0,68.0]
cT stage		
T1 T2 T3	3 (4.9%) 43 (70.5%) 15 (24.6%)	1 (3.7%) 24 (88.9%) 2 (7.4%)
Ki-67 (%)		
≤34 >34	26 (42.6%)	5 (18.5%)
	35 (57.4%)	22 (81.5%)
Grade		
1&2 3	22 (36.1%)	3 (11.1%)
	39 (63.9%)	24 (88.9%)
NLR ^2^		
≤1.909 >1.909	16 (26.2%) 45 (73.8%)	13 (48.1%) 14 (51.9%)
PLR ^3^		
≤148.14	40 (65.6%)	10 (37.0%)
>148.14	21 (34.4%)	17 (63.0%)
NLR^2^ percentage change		
≤−0.165 >−0.165	16 (26.2%) 45 (73.8%)	12 (44.4%) 15 (55.6%)
PLR^3^ percentage change		
≤0.038	22 (36.1%)	19 (70.4%)
>0.038	39 (63.9%)	8 (29.6%)
Initial echo lesion boundary		
Echogenic halo	20 (32.8%)	20 (74.1%)
Others	41 (67.2%)	7 (25.9%)
Initial echo posterior features		
Enhancement	31 (50.8%)	16 (59.3%)
Others	30 (49.2%)	11 (40.7%)
Initial Echo H/W ^4^ ratio		
≤1.221	41 (67.2%)	12 (44.4%)
>1.221	20 (32.8%)	15 (55.6%)

^1^ SD: standard deviation. ^2^ NLR: neutrophil-to-lymphocyte ratio. ^3^ PLR: platelet-to-lymphocyte ratio. ^4^ H/W ratio: tumor height-to-width ratio.

**Table 2:** Factor predicting pCR for NAC patients analyzed by using univariate and multivariate analysis.

Factors	Univariate Analysis			Multivariate Analysis		
	OR	95% CI	*p* Value	OR	95% CI	*p* Value *
Age	0.997	0.95–1.04	0.884			
Ki-67 (%)						
≤34	1					
>34	3.269	1.16–10.97	0.034			
cT stage						
T1	1	0.2–34.87	0.663			
T2	1.674	0.03–10.22	0.506			
T3	0.4					
Grade						
1&2	1			1		
3	4.513	1.37–20.51	0.024	4.013	0.81–29.64	0.119
NLR ^1^						
≤1.482	1			1		
>1.482	0.40	0.16–1.00	0.051	8.188	0.02–0.46	0.01
PLR ^2^						
≤149.546	1			1		
>149.546	1.46	0.74–2.89	0.278	0.102	1.94–45.73	0.01
NLR^1^ percentage change						
≤−0.165	1					
>−0.165	0.444	0.17–1.15	0.094			
PLR^2^ percentage change						
≤0.038	1			1		
>0.038	0.238	0.09–0.61	0.004	0.189	0.04–0.7	0.02
Initial echo lesion boundary						
Echogenic halo	1			1		
Others	0.171	0.06–0.45	0.001	0.131	0.03–0.45	0.002
Initial echo posterior features						
Enhancement	1					
Others	0.71	0.28–1.77	0.465			
Initial Echo H/W ^3^ ratio						
≤1.221	1			1		
>1.221	2.562	1.02–6.61	0.047	4.524	1.28–18.89	0.025

^1^ NLR: neutrophil-to-lymphocyte ratio. ^2^ PLR: platelet-to-lymphocyte ratio. ^3^ H/W ratio: tumor height-to-width ratio. *: *p*-value < 0.05 was deemed statistically significant.

## Data Availability

Data available on request due to restrictions of medical record privacy. The data presented in this study are available on request from the corresponding author.
